# Comparative Study of Ectopic Lymphoid Aggregates in Sheep and Murine Models of Bleomycin-Induced Pulmonary Fibrosis

**DOI:** 10.1155/2023/1522593

**Published:** 2023-01-18

**Authors:** Udari Eshani Perera, Louise Organ, Simon G. Royce, Chrishan S. Samuel, Habtamu B. Derseh, Sasika N. V. Dewage, Emmanuel Koumoundouros, Andrew Stent, Kenneth J. Snibson

**Affiliations:** ^1^School of Veterinary Science, The University of Melbourne, Parkville, VIC, Australia; ^2^Nottingham Respiratory Research Unit, University of Nottingham, Nottingham, UK; ^3^Cardiovascular Disease Program, Monash Biomedicine Discovery Institute, Department of Pharmacology, Monash University, Clayton, Victoria, Australia; ^4^Walter and Eliza Hall Institute of Medical Research, Parkville, Australia; ^5^Department of Electrical and Electronic Engineering, The University of Melbourne, Parkville, VIC, Australia; ^6^School of Veterinary Science, The University of Melbourne, Werribee, VIC, Australia

## Abstract

Idiopathic pulmonary fibrosis (IPF) is a chronic disease characterized by excessive deposition of extracellular matrix in the interstitial lung parenchyma, often manifested by dyspnea and progressive loss of lung function. The role of inflammation in the pathogenesis of IPF is not well understood. This study evaluated the histopathological and inflammatory components of bleomycin-induced pulmonary fibrosis in mouse and sheep models, in terms of their ability to translate to the human IPF. Merino sheep (*n* = 8) were bronchoscopically administered with two bleomycin infusions, two weeks apart, into a caudal lung segment, with a saline (control) administered into a caudal segment in the opposite lung. Balb/c mice were twice intranasally instilled, one week apart, with either bleomycin (*n* = 7); or saline (control, *n* = 7). Lung samples were taken for the histopathological assessment 28 days in sheep and 21 days in mice after the first bleomycin administration. We observed tertiary lymphoid aggregates, in the fibrotic lung parenchyma of sheep, but not in mouse lung tissues exposed to bleomycin. B-cell and T-cell infiltration significantly increased in sheep lung tissues compared to mouse lung tissues due to bleomycin injury. Statistical analysis showed that the fibrotic score, fibrotic fraction, and tissue fraction significantly increased in sheep lung tissues compared to murine lung tissues. The presence of tertiary lymphoid aggregates in the lung parenchyma and increased infiltration of T-cells and B-cells, in the sheep model, may be useful for the future study of the underlying inflammatory disease mechanisms in the lung parenchyma of IPF patients.

## 1. Introduction

Idiopathic pulmonary fibrosis is a chronic progressive disease characterized by the excessive deposition of the extracellular matrix (ECM) that effaces lung tissue architecture [1–4]. The disease is more common amongst males and those aged above 55–60 years [5]. The average survival time lies between 3–5 years after diagnosis [6, 7]. The pathogenesis of the disease is poorly understood due to its unknown etiology. While current treatments effectively retard the progression of the disease, thus far there has been no definitive cure for this condition [1, 2, 4].

Inflammation is often concomitant with fibrosis in the lungs of IPF patients, but the precise role of inflammation in the initiation and progression of fibrosis is unclear [8, 9]. Even though prefibrotic inflammation is not considered as a principal triggering mechanism for IPF, the reported presence of mild to moderate inflammatory cell infiltration, together with high levels of proinflammatory cytokines, chemokines, and cell surface molecules in the lung parenchyma and bronchoalveolar lavage fluids of IPF patients, suggests a link between the immune system and fibrosis [3, 9].

One interesting characteristic arrangement of immune cells in the histopathology of IPF patients is the presence of ectopic lymphoid aggregations which have been observed in the lung parenchyma [3, 9, 10]. Ectopic lymphoid aggregates are composed of dense central clusters of B-cells surrounded by T-cells in the periphery. Despite the existence of these tertiary lymphoid aggregates, it has not been determined what role they play in the underlying mechanisms of IPF [3, 9, 11].

Animal models play an important role in studying the underlying pathogenesis of IPF, and they are often used to identify potential therapeutic targets that can be exploited to treat the disease [12–15]. A variety of mouse models have been frequently used over the decades to elucidate the mechanisms involved in the disease pathogenesis of IPF [12, 15–17]. Whilst these animal models have been useful preclinical models, they do not fully replicate all aspects of human IPF [15]. We recently developed a novel bleomycin sheep model for pulmonary fibrosis that shares some characteristic features of human IPF [18]. A comparison between the sheep and mouse models with respect to pathology and inflammation has never been performed. In the present study, we compare the disease characteristics of bleomycin-induced lung fibrosis; in particular, we systematically assess the relative attributes of the histopathology of the two animal models in terms of their ability to translate to the human disease. Establishing a more accurate and representative animal model will potentially provide a more complete understanding of lung fibrosis induced by bleomycin and how it relates to the human IPF condition.

## 2. Methods

### 2.1. Experimental Design

Sheep lung tissue samples were obtained from another study [12], and the experimental design is briefly given as follows. Healthy female merino sheep (*n* = 8) aged between 9–12 months were used in the present study to induce pulmonary fibrosis using bleomycin. The experimental procedures and tissue sample collections were approved by Animal Experimentation Ethics Committee, from the University of Melbourne (Parkville, VIC, Australia), which adheres to the ARRIVE guidelines and the Australian Code of Practice for the Care and Use of Laboratory Animals for Scientific Purposes.

Similarly, female Balb/c mice (*n* = 14) aged 6–8 weeks (a comparable age to that of the sheep used, relative to the lifespan of each respective species) were utilized to compare bleomycin-induced pulmonary fibrosis to the novel sheep model established. The mouse model was established in the Department of Pharmacology, Monash University Clayton, VIC, Australia, and the studies carried out were approved by the Monash University Animal Ethics Committee, which adheres to the ARRIVE guidelines and Australian Code of Conduct for the Care and Use of Animals for Scientific Purposes.

### 2.2. Bleomycin Administration Protocol

#### 2.2.1. Sheep Model

**Figure 1:**
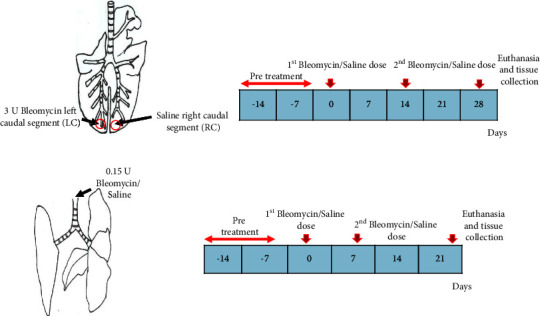
The schematic diagram of bleomycin infusion protocols in sheep (*n* = 8) and mouse (*n* = 14) models. (a) In individual sheep (*n* = 8), the left caudal lung segment received infusions of bleomycin, while the right caudal lung segment received the saline for controls. These infusions were repeated on day 14, and tissue samples were harvested on day 28. (b) For mice, there were two groups: one group of 7 animals (*n* = 7) received bleomycin intranasally, and for controls, another group of 7 (*n* = 7) received saline intranasally. These applications were repeated on day 7, and tissue samples were harvested on day 21. All the animals were kept for a 14-day resting period (pretreatment) prior to bleomycin administration.

The experimental protocol and collection of sheep tissues have been detailed previously [12–15]. Upon arrival at the animal house, the sheep were kept for a 14-day resting period (pretreatment) to acclimatize to the new environment before bleomycin administration. Following the resting period, two bleomycin doses were given 14 days apart to induce fibrosis in sheep lung segments [12–14]. This procedure involved infusing 5 ml of 0.6 U bleomycin/ml saline (a total of 3 U bleomycin) and 5 ml of 0.9% sterile saline (control) via a bronchoscope biopsy port into targeted left and right caudal lung segments of each animal (*n* = 8), respectively (Figure 1(a)). The bleomycin dose was repeated in the same manner, in all sheep, 14 days later (Figure 1(a)). All sheep were then euthanized at day 28 following exposure to barbiturate (Lethabarb) [12–14]. Targeted lung segments were identified and dissected free during the necropsy, and the main airway was cannulated to inflate the lung segment of interests. A mixture of 1 : 1 optimal cutting temperature (OCT) compound and sterile PBS solution was injected to inflate the lung segment under the pressure of approximately 20 cm/H_2_O. This was performed to preserve the tissue architecture and morphology of the lung during processing. Serial transverse sections were collected from the inflated segments (less than 0.5 cm thick) and fixed in 4% paraformaldehyde followed by 70% ethanol and processed in paraffin for histopathological analysis.

#### 2.2.2. Mouse Model

Fourteen mice were randomly divided into two groups: seven mice were treated intranasally with 0.15 U bleomycin sulphate (Hospira Healthcare Corp., Melbourne, Victoria, Australia)/animal, while the control group (*n* = 7) was treated with an equal volume of 0.9% sterile saline [16].

An equivalent dose of bleomycin or saline was given 7 days after the 1^st^ bleomycin dose, and mice were euthanized at day 21 (Figure 1(b)) via exsanguination under general anesthesia. Transverse sections of lung tissue samples (less than 0.5 cm) were collected at three different levels (proximal, middle, and distal) to the long axis during necropsy due to uneven distribution of bleomycin in each lung lobe.

Then, the collected samples were fixed in 4% paraformaldehyde followed by 70% ethanol for histopathological analysis.

### 2.3. Histopathological Examination

Paraffin-embedded tissue sections (5 *μ*m thick) were stained with haematoxylin and eosin Y (H & E) for general histological assessment of pathological changes. Collagen deposition in the lung parenchyma was measured by staining the paraffin sections with Masson's trichrome stain (trichrome stain kit, Abcam (ab150686)) according to the manufacturer's instructions.

Morphological changes in the lung tissue sections were assessed semiquantitatively and quantitatively as follows.

### 2.4. Scoring Fibrosis

Fibrosis was assessed semiquantitatively in the lung tissue sections of sheep and mice according to the scale initially proposed by Ashcroft et al. [17] in which the grades were later standardized by Hubner et al. [18]. H&E-stained lung tissue sections were evaluated by capturing images of, 10 random representatives, nonoverlapping fields under ×20 magnification. The images were then graded based on the scoring criteria outlined by Hubner et al. [18]. Lung tissues were graded from 0 to 8. Healthy lung tissues with preserved normal architecture were graded as 0. The microscopic lung tissue field completely obliterated with the fibrotic tissue mass which was graded as 8.

### 2.5. Evaluation of Inflammation

Inflammation in the lung tissue sections was graded according to Table 1. Inflammatory changes were graded for three anatomic locations: perivascular region, peribronchiolar region, and alveolar parenchyma. Images were captured as for fibrosis scoring and then scored according to the standards without knowledge of treatment.

All the images taken from samples for scoring were double-blinded and performed by an experienced veterinary pathologist AS.

### 2.6. Fibrosis Fraction

Collagen deposition in the lung parenchyma was measured from the lung tissue sections stained with Masson's trichrome stain. Ten representative, randomly selected, nonoverlapping fields with less airways and blood vessels were captured under ×20 magnification. The images were then analyzed using computer software Image Pro Plus (Version 6.3.0.512 for Windows, Media Cybernetics, Rockville, Maryland, USA). The colour selector tool was used to measure the area of tissue stained with blue (representing extracellular matrix components, of which the primary component was collagen) within the field. The fraction was obtained by dividing the blue colour-stained tissue area by the total area of the field and expressed as means and standard errors of mean (mean ± SEM).

### 2.7. Tissue Fraction

Alveolar wall thickness increases due to fibrosis and interstitial edema and can impair functional gas exchange. We captured images of ten representative randomly selected areas from each lung tissue section stained with H & E, lacking blood vessels and airways under ×20 magnification. Stained lung tissue was measured using Image Pro Plus as mentioned previously. Lung tissue fraction was calculated by dividing the tissue area by the area of the constant field of interest.

### 2.8. Evaluation of the Immune Cell Infiltration

In this study, we evaluated the infiltration levels of B-cells and T-cells in sheep and mouse lung tissues during bleomycin-induced pulmonary fibrosis. Immunohistochemistry was performed using the biomarkers, paired box-5 (Pax-5), and Cluster of Differentiation-3 (CD-3) to identify B-cells and T-cells, respectively. Each assay was performed with a negative control and a sheep lymph node and a mouse thymus for positive controls.

### 2.9. Evaluation of B-Cell Infiltration

Immunohistochemistry was performed using the Monoclonal Mouse Anti-HumanPax-5 primary antibody (BD Biosciences, USA) to evaluate B-cell infiltration levels in sheep and mouse lung tissues. Paraffin sections were dewaxed in three changes of xylene 5 min each and rehydrated in two changes of absolute ethanol for 5 min each followed by 70% ethanol for 5 min. Antigen retrieval was performed using preheated citrate buffer, pH-6 heated for 15 min, before the slides were left to cool down for 10 min, followed by PBS washing. The slides were incubated in 3% H_2_O_2_ for 10 min to block endogenous peroxidases and then rinsed thoroughly with PBS. Undiluted fetal calf serum (FCS) was added to each slide and incubated for 1 hour to block nonspecific antigen binding. The anti-Pax-5 antibody was diluted 1 : 4 with FCS and applied to the tissue sections for 1 hour of incubation. The slides were rinsed gently with PBS. EnVison dual link system-HRP (Horseradish Peroxidase) (Dako, North America Inc., CA, USA) was applied and incubated for 30 min. To visualize the antigen-antibody reaction, NovaRED peroxidase substrate (Vector Laboratories Inc., CA, USA) was added to each sample and incubated for 3 min. Then, the samples were washed with distilled water to stop the reaction and counter-stained with haematoxylin.

### 2.10. Evaluation of T-Cell Infiltration

Immunohistochemistry was performed using a Polyclonal Rabbit Anti-Human CD-3, Ready-to-Use antibody (Dako, North American Inc., USA) as mentioned previously to identify T-cells present in the bleomycin/saline-infused sheep and mouse lung tissues.

### 2.11. Quantitative Image Analysis

Images were captured using a Leica DM500 microscope. Twenty representative nonoverlapping fields in the lung parenchyma were captured from each lung tissue sections under ×40 magnification. The numbers of red colour-stained B-cells and T-cells that were present in each field of lung parenchyma were counted, and the values were expressed as the mean ± standard error of the mean (mean ± SEM). Tertiary lymphoid aggregates were counted and then standardized to 1 cm^2^ of lung tissue.

### 2.12. Statistical Analysis

Statistical analysis was performed using GraphPad Prism software, version 8.0.1 for Windows (GraphPad Software, La Jolla California, USA). The degree of fibrosis and inflammation was analyzed using the Mann–Whitney test. Immune cell infiltration levels, fibrotic fraction, and the tissue fraction were evaluated using a one-way ANOVA, with Tukey's post-hoc test to make multiple comparisons between the groups. The data were expressed as the mean ± standard error of the mean (mean ± SEM). A *p* value of less than 0.05 (*p* < 0.05) was considered as statistically significant.

## 3. Results

### 3.1. Comparison of Histopathological Attributes of the Two Animal Models in Response to Bleomycin Injury

**Figure 2:**
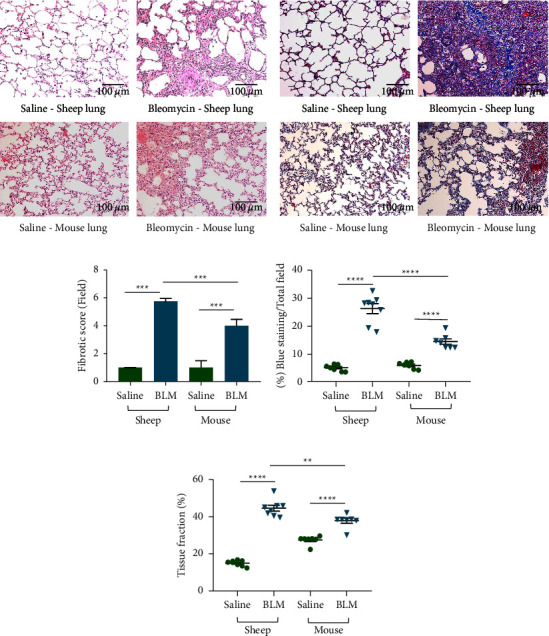
Fibrotic scores, fibrotic fractions, and tissue fractions of sheep (*n* = 8) and mouse (*n* = 14) lung tissues administered with either saline (control) or bleomycin (treatment). (a) H&E staining was used to evaluate the fibrotic score and tissue fraction. (b) Masson's trichrome staining was used to evaluate the % of blue staining. Ten representative randomly selected areas from each lung tissue sections were captured under ×20 magnification for the analysis. Fibrotic scores (c) were expressed as a median and interquartile range while fibrotic fraction (d) and tissue fraction (e) were expressed as means and standard errors of means. Significance was determined using the Mann–Whitney test and one-way ANOVA denoted as follows: ^*∗∗*^*p* < 0.005, ^*∗∗∗*^*p* < 0.0005, and ^*∗∗∗∗*^*p* < 0.0001.

The severity and distribution of fibrosis were evaluated in both sheep and mouse lung tissue sections (Figure 2). Moderate to severe irregular, well-demarcated multifocal collagen deposits were observed in the lung parenchyma and peribronchial regions of bleomycin-infused sheep lung segments. Excessive collagen deposition resulted in irregular thickening of alveolar septa in sheep lung tissue. This multifocal fibrosis imparted a heterogeneous appearance to sheep lung tissues. In contrast, the lung tissue architecture of the saline-infused lung segments of sheep displayed minimal fibrotic changes, consistent with healthy lung tissue (Figures 2(a) and2(b)).

In mouse lung tissues, moderate collagen deposition was observed in the peribronchial regions and mild to moderate irregular multifocal fibrosis was observed in the lung parenchyma at the fibrotic stage. As expected, no fibrotic changes were observed in the lung tissue architecture of saline-infused control mice, consistent with normal healthy lungs (Figures 2(a) and 2(b)).

Both sheep and mouse models showed a statistically significant increase in fibrotic scores in lung tissues with bleomycin infusions compared to saline infusions (bleomycin 5.75 vs. saline 1.00; *p* < 0.0001 in sheep and bleomycin 4.00 vs. saline 1.00; *p* < 0.0001 in mice). Significantly higher fibrotic scores were observed in bleomycin-infused sheep lung segments compared to bleomycin-treated mouse lungs (5.75 vs. 4.00; *p* < 0.0001) (Figure 2(c)).

To further support the previous findings, we quantitatively evaluated the collagen and connective tissue deposition in sheep and mouse lung tissue sections stained blue with Masson's trichrome. The percentage of blue staining increased significantly in both sheep (bleomycin 26.50 ± 1.77% vs. saline 5.45 ± 0.39%; *p* < 0.0001) and mouse (bleomycin 14.77 ± 0.95% vs. saline 6.26 ± 0.43%; *p* < 0.0001) lung tissues as a response to bleomycin infusion (Figure 2(d)). Comparison of the percentage of blue staining (fibrotic fraction) between the two models revealed that the sheep lung had a higher fibrotic fraction after bleomycin treatment (sheep 26.50 ± 1.77% vs. mice 14.77 ± 0.95%; *p* < 0.0001) (Figure 2(d)).

Then, we determined the tissue fraction in both species to evaluate the functional status of the lung tissues. Bleomycin infusion significantly increased the lung tissue fraction in both sheep (bleomycin 44.91 ± 1.57% vs. saline 15.29 ± 0.53%; *p* < 0.0001) and mouse (bleomycin 38.16 ± 1.37% vs. saline 27.76 ± 0.88%; *p* < 0.0001) models (Figure 2(e)). This was mainly due to the excessive deposition of the collagen fibres in the alveolar septa and the pulmonary interstitial edema. We compared the tissue fraction between the two models, and it was significantly increased in sheep lung tissues when compared to mouse lung tissues (sheep 44.91 ± 1.57% vs. mice 38.16 ± 1.37%; *p* < 0.002) (Figure 2(e)).

### 3.2. Evaluation of Inflammation in Animal Models When Modelling Pulmonary Fibrosis

Inflammation was evaluated in 3 anatomical regions: alveolar, perivascular, and peribronchial regions of the sheep and mouse lung tissues.

**Figure 3:**
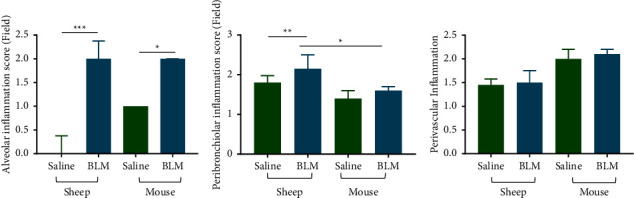
Alveolar inflammation (a), peribronchiolar (b), and perivascular (c) inflammation of sheep (*n* = 8) and mouse (*n* = 14) lung tissues infused with either saline (control) or bleomycin (treatment). The data were evaluated for tissue sections stained with H&E. Ten representative randomly selected areas from each lung tissue section were captured under ×20 magnification for the analysis. All the data were expressed as the median and interquartile range. Significance was determined using the Mann–Whitney test and denoted as follows: ^*∗*^*p* < 0.05, ^*∗∗*^*p* < 0.005, and ^*∗∗∗*^*p* < 0.0005.

Alveolar inflammation significantly increased in both sheep and mouse lung tissues in response to infusions of bleomycin (bleomycin 2.00 vs. saline 0.00; *p* < 0.0001 in sheep and bleomycin 2.00 vs. saline 1.00; *p*=0.01 in mice (Figure 3(a)). However, alveolar inflammation did not vary between the two species (Figure 3(a)). Peribronchiolar inflammation significantly increased in sheep lung segments in response to the bleomycin infusion, while the small increase in peribronchiolar inflammation in the mouse lung after bleomycin was not statistically significant (bleomycin 2.15 vs. saline 1.80; *p*=0.02 for sheep and bleomycin 1.60 vs. saline 1.40; *p*=0.24 for mice) (Figure 3(b)). When comparing the two bleomycin models, there was a significant increase in the peribronchiolar inflammation in the sheep model (2.15 for sheep vs. 1.60 for mice; *p*=0.002) (Figure 3(b)). Perivascular inflammation was not significantly different between bleomycin and saline lung tissues for both sheep and mice (bleomycin 1.50 vs. saline 1.45; *p*=0.3 in sheep and bleomycin 2.10 vs. saline 2.00; *p*=0.7 in mice (Figure 3(c)).

### 3.3. Immune Cell Infiltration Varied in Sheep and Mouse Lung Tissues

We evaluated the B-cell and T-cell infiltration and distribution patterns in sheep and mouse lung tissue sections taken from postmortem samples.

**Figure 4:**
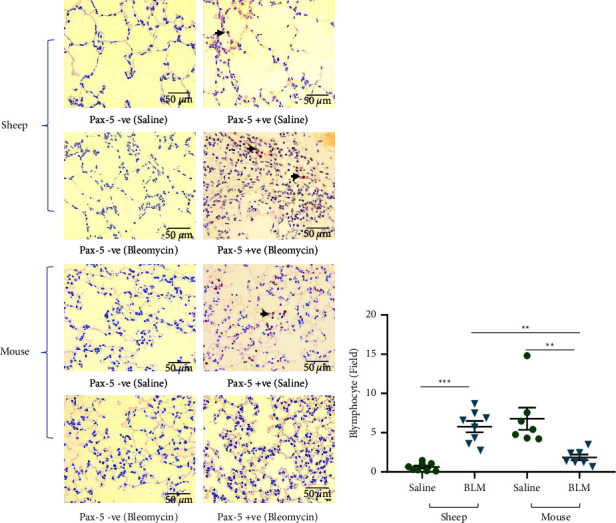
Immunohistochemical staining of B lymphocytes (PAX-5 +ve) in sheep and mouse parenchymal lung tissues (a). Arrows indicate B lymphocytes. The graph represents the B-cell infiltration in saline-infused and bleomycin-infused lung segments of sheep (*n* = 8) and lung tissues from saline control (*n* = 7) and bleomycin-treated mice (*n* = 7) (b). Twenty representative nonoverlapping fields in the lung parenchyma were captured from each lung tissue section under ×40 magnification for analysis. Each bar represents the mean ± standard error of the mean. Significance was determined by one-way ANOVA and Tukey's post-hoc test to make multiple comparisons test between the groups. ^*∗*^*p* < 0.05, ^*∗∗*^*p* < 0.005, and ^*∗∗∗*^*p* < 0.0005.

There were marked differences between sheep and mice in terms of B-cell infiltration into lung tissues (Figure 4(a)). While mild B-cell infiltration was present in sheep lung tissue parenchyma in the saline control lung segments, B-cell infiltration was significantly increased in bleomycin-infused lung segments (bleomycin 5.76 ± 0.72 vs. saline 0.61 ± 0.17 no. of B-cells/field; *p* < 0.0001) (Figure 4(b)). In contrast, while moderate, diffuse B-cell infiltration was observed in the control mouse lung tissues, there were significantly less parenchymal B-cells in bleomycin-exposed mouse lung tissues when compared with lung samples from saline control mice (bleomycin 1.92 ± 0.42 vs. saline 7.20 ± 1.59 no. of B-cells/field; *p*=0.001) (Figure 4(b)).

The B-cell infiltration was significantly higher in bleomycin-exposed sheep lung tissues when compared with mouse tissues (sheep 5.76 ± 0.72 vs. mice 1.92 ± 0.42 no. of B-cells/field; *p* < 0.05) (Figure 4(b)).

**Figure 5:**
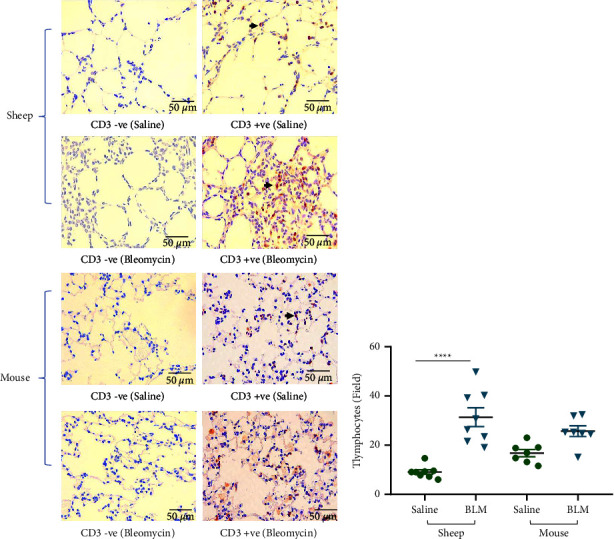
Immunohistochemical staining of T lymphocytes (CD-3 +ve) in sheep and mouse lung tissues (a). Arrows indicate examples of T lymphocytes. The graph represents the T-cell infiltration levels between saline-infused and bleomycin-infused lung segments of sheep (*n* = 8) and lung tissues from saline control (*n* = 7) and bleomycin-treated mice (*n* = 7) (b). Twenty representative nonoverlapping fields in the lung parenchyma were captured under ×40 magnification for analysis. Each bar represents the mean ± standard error of the mean. Significance was determined by one-way ANOVA and Tukey's post-hoc test to make multiple comparisons test between the groups. ^*∗∗∗∗*^*p* < 0.0001.

T-cell infiltration varied in both species. In sheep, mild, diffuse T-cell infiltration was observed in the lung parenchyma of control lung segments (Figure 5(a)). The level of T-cell infiltration was markedly increased in the lung parenchyma of bleomycin-infused lung segments of sheep (bleomycin 31.3 ± 3.8 vs. saline 9.06 ± 0.9 no. of T-cells/field; *p* < 0.0001) (Figure 5(b)). In addition, T-cell populations which surrounded tertiary lymphoid aggregates were often seen in the sheep lung parenchyma.

In mice, the control group displayed mild to moderate, diffuse, T-cell infiltration in the lung parenchyma, which did not significantly increase in the parenchyma of mice treated with bleomycin (bleomycin 25.75 ± 2.5 vs. saline 17.34 ± 1.6 no. of T-cells/field; *p*=0.17) (Figure 5(b)).

**Figure 6:**
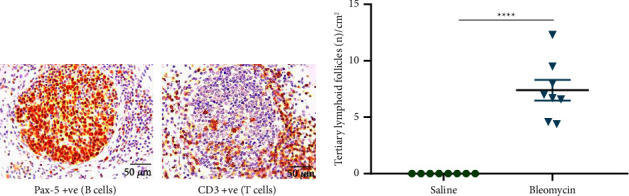
Tertiary lymphoid aggregates in the parenchyma of sheep lung segments exposed to bleomycin. The top two panels show serial sections immuno-stained with either a Pax-5 primary antibody to identify B-cells or a CD-3 antibody to identify T-cells (a) (40x). The graph shows the number of tertiary lymphoid aggregates in the lung parenchyma of saline and bleomycin-infused lung segments of sheep (*n* = 8) (b). Note that zero lymphoid aggregates were found in saline control lung segments (*n* = 8). Each bar represents the mean ± standard error of the mean. Significance was determined using the *t* -test and denoted as follows: ^*∗∗∗∗*^*p* < 0.0001.

An interesting observation from the lymphocyte analyses performed in Figures 4 and 5 was that in addition to the diffuse lymphocytic infiltration observed, we also noted tertiary lymphoid aggregates, or ectopic lymphoid follicles, in the sheep lung parenchyma in response to bleomycin. These tertiary lymphoid aggregates were variable in size and ranged from 0.018 to 0.046 mm^2^. The tertiary lymphoid follicles were well-formed B cell germinal centres surrounded by T-cell aggregates towards the periphery (Figure 6(a)). Interestingly, we did not observe any tertiary follicular structures in the lung parenchyma of the control lung segments in sheep, and their presence was not observed in any of the parenchymal lung tissues taken from both groups of mice.

To evaluate the number of tertiary lymphoid follicles formed in the bleomycin-treated sheep lung, we counted the number of follicles that were present in the lung parenchyma. A significant increase in the number of tertiary lymphoid follicles was observed in response to bleomycin in the sheep lung parenchyma (bleomycin 7.38 ± 0.91 vs. saline 0 ± 0 no. of B lymphocytic aggregations/cm^2^; *p* < 0.0001) (Figure 6(b)).

## 4. Discussion

This study compared the histopathological and inflammatory features of sheep and mouse models of bleomycin-induced pulmonary fibrosis. A major finding of this study was that we found the presence of tertiary lymphoid follicles in bleomycin-treated sheep lung tissues. This study is the first to report that tertiary lymphoid follicles form in response to bleomycin in the lung tissues of sheep, and interestingly, these aggregates share many similar characteristic features of the lymphoid aggregates described from IPF patients [3, 9, 10].

Surprisingly, we did not observe tertiary/ectopic aggregates in mouse lung parenchyma after bleomycin exposure. This may be possibly due to structural/tissue differences between large animals (human/sheep) and small animals (mice rats). These differences may mean that animals respond slightly differently to lung damage episodes.

In previous studies, lymphoid aggregates have been observed in the submucosa around the airways and blood vessels of mice, but they were not found in the mouse lung parenchyma [19–21]. The lymphoid aggregates around the airways and blood vessels in the aforementioned studies were induced under a variety of different experimental conditions in mice [19–21]. In our study, tertiary lymphoid aggregates were not found and B-cell infiltration into mouse lung parenchyma was significantly reduced on day 21. It should be noted that in a previous study in mice, B-cell infiltration declined after 7 days of postbleomycin infusion [22], which is consistent with the low levels of B-cell infiltration found in the current study.

Tertiary lymphoid follicles are one of the characteristic histopathological findings observed in the lung parenchyma of IPF patients [3, 9, 10]. They have been classified as ectopic lymphoid follicles that can be formed due to persistent injury, inflammation, or infections [23]. However, the actual function and contribution of the tertiary lymphoid follicles to the pathogenesis of IPF have not been determined [3, 8, 10]. The two potential hypotheses are that tertiary lymphoid aggregates may emerge to enhance local immune responses and support the function of the secondary lymphoid organs or that they may exacerbate the pathology associated with chronic inflammation [24]. The formation of tertiary lymphoid aggregates is linked with inflammation driven by irritants or infections, and these tertiary lymphoid follicles can potentially serve as a site for the induction of protective local immune responses [23]. Previous studies showed that the number of tertiary lymphoid follicles increased as disease progression worsened in IPF patients [9]. The presence of these lymphoid follicles in patients with severe lung fibrosis suggests that active cellular inflammation continues in IPF even at the later stages of disease progression. Interestingly, it seems that a constant immunological stimulus is needed to maintain the presence of these lymphoid aggregates. We based this on our unpublished findings in which tertiary lymphoid aggregates were completely absent in sheep lung parenchyma sampled 16 weeks after bleomycin exposure.

The tertiary lymphoid structures in the sheep are organized with a central region of nonproliferating B-cells surrounded by an infiltrate of T-cells, consistent with the follicular structures documented in IPF patients [23, 24].

Overall, given that the function of these lymphoid aggregates in the underlying disease pathology is not known, the sheep model might be useful for investigating the functional relationship between tertiary lymphoid follicles and the lung pathology of IPF. Investigating the pathogenic significance of these follicles involved could be especially facilitated by accessing and analysing efferent lymph nodes from the lungs of sheep with thoracic duct cannulations [25].

Our findings showed a significant increase in T-cell infiltration in association with fibrosis in the sheep lung parenchyma, which is consistent with the T-cell infiltration pattern detected in IPF patients [3, 26, 27]. T-cells are known to infiltrate diffusely into the alveolar septa and interstitium of IPF patients [26, 27]. Even though there is an increase in the number of T-cell infiltration in the mouse lung parenchyma in response to bleomycin, there were no statistically significant differences between saline and bleomycin lung segments in 21 days. This may be due to the reduction of T-cell infiltration which has been shown to occur seven days after bleomycin doses in mice [21]. Interestingly, both species had T-cell aggregates in peribronchial regions in response to airway administration of bleomycin [19, 28].

We found that sheep lung segments had higher fibrotic scores and fibrotic fractions when compared to mouse lungs. These higher fibrotic scores in the sheep model may have resulted from this species having a larger alveolar size, thicker alveolar walls, more collagen and elastin fibers, or a greater interstitial matrix mass in comparison to mice. The increased dimensions of these structural parameters provide greater structural support for the increased lung sizes of larger species [15, 29, 30]. Thus, it could be argued that the increased interstitial connective tissue in sheep lungs may facilitate an augmented fibrotic response compared with the lungs of smaller species. However, with this asset in mind, there are a number of factors such as cost, animal ethics, reproducibility, genetic modification, and availability of more reagents which favor the use of small animal species in lung fibrosis research [31].

While bleomycin is the most frequently used agent to induce fibrosis in animal models of pulmonary fibrosis, there are limitations that need to be acknowledged. IPF is a chronic progressive and an irreversible disease often seen in older people aged above 55 years. In both the sheep and mouse models used in this study, the age of the animals was relatively young. The choice of using juvenile animal species is mainly due to practical problems of reducing costs and the time taken to induce lung fibrosis. Although many of the pathways that are involved in the development of fibrosis in older and young animals are similar [31], the age difference between animal models and human patients potentially hampers any investigation into the mechanisms associated with the progressive nature of IPF in the aging lungs.

There is a slight difference between the two animal models in regard to the procedure for introducing bleomycin to the lungs of the animals. In sheep, the bronchoscopy procedure was used to infuse bleomycin directly to the lower airways, while for mice, the intranasal procedure was used due to the impracticability of bronchoscopy in the smaller species. Notwithstanding these small procedural differences, bleomycin was still delivered to the parenchymal lungs via the airways in both models. Furthermore, airway delivery of bleomycin made the comparison of disease parameters between the species more relevant than if we used either the intraperitoneal, oral, or parenteral routes of administration in mice.

There are a number of animal models developed to study human IPF, and each model has its own attributes. It is appropriate to select a suitable model accordingly to obtain the more representative outcome.

## 5. Conclusion

This study provides a comparative description of the pathology and inflammatory features associated with sheep and mouse models of bleomycin-induced pulmonary fibrosis. Though there were many similarities detected between the two models, the unique presence of tertiary lymphoid aggregates in the lung parenchyma, and increased infiltration of T-cells and B-cells, suggests that the sheep model could be useful for studying these parameters and how they relate to the underlying disease mechanisms of human IPF.

## Figures and Tables

**Table 1:** Inflammation scores for the assessment of lung tissue damage.

Inflammation scores	
*Perivascular inflammation scores*	
1+	Mild infiltration of inflammatory cells
2+	Moderate infiltration of inflammatory cells
3+	Heavy infiltration of inflammatory cells

*Peribronchiolar inflammation scores*	
1+	Mild infiltration of inflammatory cells
2+	Moderate infiltration of inflammatory cells
3+	Heavy infiltration of inflammatory cells

*Alveolar inflammation scores*	
0	No inflammation
1+	Mild increase in inflammatory cells
2+	Moderate patchy increase in inflammatory cells
3+	Moderate patchy increase in inflammatory cells and patchy consolidation of alveolar parenchyma
4+	Severe confluent consolidation of alveolar parenchyma

## Data Availability

The raw datasets in this study can be obtained from the corresponding author upon request.

## Abbreviations

IPF: Idiopathic pulmonary fibrosis
OCT: Optimal cutting temperature
LC: Left caudal
RC: Right caudal
BLM: Bleomycin
PaX-5: Paired box-5
CD-3: Cluster of differentiation-3.
