# Correction to “Comparative Study of Ectopic Lymphoid Aggregates in Sheep and Murine Models of Bleomycin‐Induced Pulmonary Fibrosis”

**DOI:** 10.1155/carj/9862083

**Published:** 2026-08-24

U. E. Perera, L. Organ, S. G. Royce, et al., “Comparative Study of Ectopic Lymphoid Aggregates in Sheep and Murine Models of Bleomycin‐Induced Pulmonary Fibrosis,” *Canadian Respiratory Journal* 2023, no. 1 (2023): 1–11, https://doi.org/10.1155/2023/1522593.

In the article titled “Comparative Study of Ectopic Lymphoid Aggregates in Sheep and Murine Models of Bleomycin‐Induced Pulmonary Fibrosis,” there is an error in Figure 5. An image was inadvertently duplicated from Figure 4 (a) Mouse Pax‐5 ‐ve (Saline) into Figure 5 (A) Mouse CD3 ‐ve (Bleomycin). This error occurred during figure assembly.

Figure 5 is corrected as follows:

**Figure 5:**
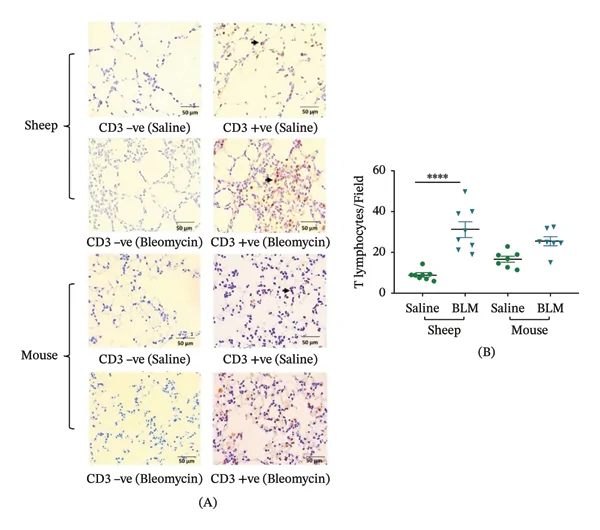
Immunohistochemical staining of T lymphocytes (CD‐3 +ve) in sheep and mouse lung tissues (A). Arrows indicate examples of T lymphocytes. Graph represents the T‐cell infiltration levels between saline‐ and bleomycin‐infused lung segments of sheep (*n* = 8) and lung tissues from saline control (*n* = 7) and bleomycin‐treated mice (*n* = 7) (B). Twenty representative non‐overlapping fields in the lung parenchyma were captured under × 40 magnification for analysis. Each bar represents the mean ± standard error of the mean. Significance was determined by one‐way ANOVA and Tukey’s post hoc test to make multiple comparisons test between the groups.  ^∗∗∗∗^ *p* < 0.0001.

We apologize for this error.

